# Adolescent Pregnancy and Challenges in Kenyan Context: Perspectives from Multiple Community Stakeholders

**DOI:** 10.1007/s40609-017-0102-8

**Published:** 2017-10-25

**Authors:** Manasi Kumar, Keng-Yen Huang, Caleb Othieno, Dalton Wamalwa, Beatrice Madeghe, Judith Osok, Simon Njuguna Kahonge, Joyce Nato, Mary McKernon McKay

**Affiliations:** 1 Department of Psychiatry, University of Nairobi, PO Box 47074, Nairobi 00100, Kenya; 2 Department of Public Health and Child and Adolescent Psychiatry, New York University, New York, NY 10016, USA; 3 Department of Pediatrics, University of Nairobi, PO Box 47074, Nairobi 00100, Kenya; 4 Kenya Medical Training College, PO 3010095, Nairobi, Kenya; 5 Mental Health and Substance Use Management (MHSM) Unit Secretariat, Kenya Board of Mental Health, Ministry of Health, Nairobi, Kenya; 6 Noncommunicable Diseases, WHO, Nairobi, Kenya; 7 George Warren Brown School of Social Work, Washington University in St. Louis, St. Louis, MO, USA

**Keywords:** Pregnant adolescent in Nairobi, Stigma, Community support, Food insecurity, Primary health care facility

## Abstract

**Objective:**

The key objective of this paper is to provide a phenomenological account of the mental health challenges and experiences of adolescent new mothers. We explore the role of social support and the absence of empathy plays in depression among pregnant adolescents. The project also collected data on the adolescents’ caregiving environment which includes the adolescents’ mothers, their partners, the community, and health care workers, as well as feedback from staff nurses at the maternal and child health centers. The caregivers provide additional insight into some of the barriers to access of mental health services and pregnancy care, and the etiology of adolescents’ distress.

**Methods:**

The interviews were conducted in two health facilities of Kariobangi and Kangemi’s maternal and child health (MCH) centers that cover a huge low-income and low-middle-income formal and informal settlements of Nairobi. A grounded theory approach provided a unique methodology to facilitate discussion around adolescent pregnancy and depression among the adolescents and their caregivers. Our interviews were cut across four samples with 36 participants in total. The sample 1 comprised of eight pregnant adolescents who screened positive for depression in Kariobangi, sample 2 were six caregivers from both sites, and sample 3 were 22 new adolescent mothers from both sites. After individual interviews, we carried out one focused group discussion (FDG) in order to understand the cross-cutting issues and to gather some consensus on key issues, and the sample 4 were 20 community health workers, health workers, and nurses from both sites. We had one FGD with all health facility-based workers to understand the cross-cutting issues. The interviews in sample 1 and 2 were individual interviews with pregnant and parenting adolescents, and their caregivers. All our adolescent participants interviewed in sample 1 were screened for depression. Individual interviews followed the FGD.

**Findings:**

Pregnant and parenting adolescents faced several adversities such as social stigma, lack of emotional support, poor healthcare access, and stresses around new life adjustments. We highlighted a few useful coping mechanisms and strategies that these adolescents were thinking to reduce their stress. Primary social support for pregnant and parenting teens comes from the adolescent’s mother. The external family and male partners provide negligible support in the rearing of the child. While the mother’s reactions to the daughters’ pregnancy were empathetic sometimes, absence of food and resources made the mother distant and constraint in lending support. For those adolescents who were living with partners, in their new mother role, they had to negotiate additional challenges such as solutions to everyday childcare responsibilities and other family duties. The health care workers and community health workers confirmed that adolescent mothers have multiple needs, but there is a lack of holistic approach of service, and that their own training and capacities were very limited.

**Conclusions:**

Our paper highlights several individual stakeholder-related and system-level barriers in the MCH primary care setting that affect delivery of psychosocial support for pregnant adolescent. We have identified these knowledge, practice, and institutional gaps that need addressing through careful community and health service staff engagement using implementation strategies that are effective in low-resource settings. Pregnant adolescents are highly vulnerable group and mental health services needs to be understood better.

## Introduction

Adolescent pregnancy is a significant public health problem in low- and middle-income countries (LMICs) (WHO 2014). Worldwide, it is estimated that around 16 million girls aged 15 to 19 or 1 million girls under 15 give birth every year (accounting for 11% of birth worldwide) (UNFPA 2013). Additionally, babies born to adolescent mothers have a much higher likelihood of dying and are exposed to other life-threatening conditions (Mori et al. 2013; WHO 2014). Adolescent pregnancy and pregnancy complications during childbirth are the major contributors to mortality among 15–19-year-old adolescents and young women (UNFPA 2008, 2013). Child mortality in LMICs, lack of resources, and poverty add to the cycle of poor health (WHO 2014). We have a number of findings on physical health-related challenges associated with adolescent pregnancy; however, the role of mental health and psychological well-being among this group has not been well studied in Kenya. Addressing adolescent pregnancy and well-being of the mothers and children is a top priority in global public health, and the need to identify the barriers to the uptake of mental health care is vital to our understanding of their challenges. This study explores multiple stakeholders’ perspectives, primarily among pregnant and parenting adolescents around the issues related to adolescent’s pregnancy within the Kenyan context. Adolescence typically refers to ages from 10 to 24 years of age with early, middle, and late adolescents representing different developmental stages within the broad band (UNICEF 2016). Parenting adolescents or young mothers in the paper consequently are young people within this broad band who are parents themselves before reaching the age of 20 (referring to the phenomenon of “children having children”).

## Global Health Research Overview in Pregnant Adolescents

Women’s equitable access to optimal health and educational opportunities is key to physical, social, and economic well-being of families and societies. Global Strategy for Women’s and Children’s Health launched by the UN Secretary General (Ki-moon 2015) stresses the importance of addressing the health and welfare of adolescent girls, in addressing adolescent morbidity and mortality in “multi-burden countries” (Patton et al. 2016). Adolescent pregnancy and underage motherhood present serious concerns for the mother, baby, community, and society at large (Sawyer et al. 2012; Ramirez et al. 2013). Research has documented that younger age, poor social or family support, unstable relationships, and inadequate access to health facilities or to educational opportunities push these adolescents into further psychiatric and health-related morbidities with poorer outcomes for their offspring (Patel et al. 2007; Patton et al. 2016; Ki-moon 2016). Still, births and deaths in the first week of life are 50% higher among babies born to adolescent mothers than among babies born to mothers in their twenties (UNFPA 2013). The prevalence of depression is high during the perinatal period, with worldwide estimates of 11–18%, and estimates in low-income countries are as high as 30–50%. Worldwide, 16,000,000 births to 15–19-year-old adolescent annually accounts for 11% of births. About 95% of births to adolescent mothers occur in low-income countries (WHO 2008). Young mothers experience a two- to nine fold increased prevalence of perinatal mental disorders (Fisher et al. 2013). Specifically, depression, during and after pregnancy, is linked to multiple adverse health outcomes for mothers and children.

Adolescent pregnancy is one of the commonest risk factor exposing young girls to the risk of HIV and sexually transmitted infections (STIs). Girls in a marriage or union often have older, more sexually experienced husbands or partners; lack the power to negotiate safer sex; and have little access to family planning information (UNESCO 2013). The significance of adolescent mental health disorders over the lifespan has only recently caught attention and remains a relatively understudied area in global health (Patton et al. 2014). Primary health care facilities have taken on the burden of ensuring the child well-being among the local communities. Early intervention and adolescent-friendly services have caught attention in high-income countries but remain an area requiring more efforts. Evaluation studies have noted that very limited interventions have been tested to address adolescent mental health (Jorm 2015; Stockings et al. 2016), and in the few that have been used, their effectiveness for high risk or for differential cultural/socioeconomic contexts has not been demonstrated. These findings suggest that socio-culturally relevant psychosocial outreach services are urgently needed. Recommendations are for multimodal efforts to integrate schools, families, community health services, and mass media-based interventions.

Most global health research in adolescent pregnancy has focused primary on addressing maternal and child mortality, and increasing awareness of the risk of HIV/ STDs. WHO focus has been on preventing early pregnancies and reducing poor reproductive health outcomes (e.g., reducing early marriage, reducing pregnancy before the age of 20, increasing the use of contraception, reducing coerced sex, reducing the rate of unsafe abortions, and increasing the use of skilled antenatal, childbirth, and postnatal care) (WHO 2014; Patton et al. 2016). Additionally, legal rights and institutional arrangements for well-being of children and adolescents in vulnerable, high-risk contexts have been secured under UN’s Convention on the Rights of the Child (ratified by all countries of the world with the exception of the USA and Somalia).

## Pregnant and Parenting Adolescents Needs and Challenges in LMIC Contexts

Given this global picture, about 95% of births to adolescent mothers occur in low-income countries (WHO 2008). This is an issue that affects low-income countries the most, requiring urgent redressal. In Sub-Saharan Africa (SSA), about 20–40% of adolescents are mothers or currently pregnant (Gyesaw and Ankomah 2013). Only a handful of studies have specifically focused on mental health or studies with SSA populations (e.g., Atuyambe et al. 2005; Kaye 2008; Gyesaw and Ankomah 2013; Aziato et al. 2016). Given the high rates of poverty, food insecurity, domestic violence, health problems, and different health system setups in LMICs which rely solely on semi-professional or non-health professionals in maternal and child health care, it is likely that the challenges that pregnant/parenting adolescents face in LMICs will be different from other adolescents, and deserves nuanced study. The rate of adolescent pregnancies is more common in urban areas than in rural areas, with estimated prevalence rates in urban areas as high as 30% (UNESCO 2014; Pearson et al. 2013). In Kenya, 18% of young women ages 15–19 have already begun childbearing; 15% are mothers and an additional 3% are pregnant with their first child (KHDS 2008–2009). Young motherhood is slightly more common in urban areas than in rural areas, where approximately 30% of adolescent girls get pregnant in most urban centers. Most adolescent pregnancies are unplanned and more likely to occur among poor and uneducated communities (Kenya Ministry of Health 2015; Kenya National Standards for Maternal Care 2002). Adolescence is also a period of developmental transition between childhood and adulthood, and pregnancy during this period poses greater risk for young mothers and their children. Some urban slum areas in Kenya have an estimated maternal mortality rate as high as 706 deaths per 100,000 births (Guttmacher Institute 2012). Another factor unique to Kenya and the SSA context is the absence of a male partner and/or relative support once an adolescent becomes pregnant. The social stigma and shifting of responsibility from the couple (adolescent girl and her boyfriend/partner) to the girl is automatic and disturbing.

There is a paucity of systematic research to understand the causes of depression in pregnant adolescents or parenting adolescents, which have critical implications in developing needed services to address health burdens. The impact of adolescent pregnancy on mental health, especially depression, in LMICs has been well established; however, integration of mental health care and enhancing mental health services and outreach is still lacking and poorly documented for this group. While issues like task-shifting and task-sharing were proven to be effective in delivering mental health services in primary health care, reaching out to vulnerable adolescent populations has been very limited in Kenyan context (Juma et al. 2013). A review study suggested that the prevalence of perinatal depression in parenting adolescents is two to nine times higher than that in the general population, with worldwide estimated prevalence of 11–18% and 30–50% for low-income countries (Fisher et al. 2013). Atuyambe et al. (2005) conducted a qualitative study in Uganda whose findings revealed that pregnant adolescents faced domestic physical violence and were psychologically violated by parents and partners, and the community within which they lived. The respondents included pregnant adolescents, adolescent mothers, opinion leaders, persons in charge of health units, and traditional birth attendants. Similarly, another qualitative study from Uganda (Kaye 2008) explored what adolescent mothers perceive as their struggles during the period of transition from childhood to parenthood. Overall, young adolescents reported more anxiety; loss of self-esteem (when they conceived); difficulty in accessing financial, moral, and material support from parents or partners; and stigmatization by health workers when they sought care from health facilities. Another recent study suggested the causes for high prenatal depression can be attributed to violence and relationship stress (Onu et al. 2016). Pregnancies in adolescents from low-income countries pose greater challenges, especially around finances, in interpersonal relationship within families and with partners, and in accessing moral and material support (Fisher et al. 2012). Furthermore, these pregnant and parenting adolescents face additional burdens when accessing health care such as stigmatization by health workers.

Financial and interpersonal challenges are causes and consequences of perinatal depression (Atuyambe et al. 2009; van Ginneken et al. 2013). These challenges may further lead to perinatal depression and exacerbate adverse outcomes for mothers and their offspring. Perinatal depression poses high development costs for communities particularly in perpetuating the cycle of poverty (UNFPA 2013). The sustainable development goals (SDGs) document that underage pregnancies pose irreparable consequences for individuals and communities (Ki-moon 2016*). At the individual level*, many pregnant adolescents face inter-family conflict and some experience domestic physical violence. Many pregnant adolescents are often psychologically violated or stigmatized by their parents, partners, and the community in which they reside (Kaye 2008). *At the system level*, efforts for engagement and outreach with adolescent girls in order to improve their health and well-being, require inter-agency and inter-community collaboration between the school, community, and health care system in order to make services more accessible to pregnant and parenting adolescents. Furthermore, there is a need to understand implementation science issues around integrating adolescent mental health within broader social and legal institutions using an ecological framework.

### Objectives of the Paper

The current study investigates the experiences of Kenyan pregnant and parenting adolescents around pregnancy, parenting, and depression, with the goal of identifying the needs and challenges faced by adolescents from LMICs. The key objective of the paper is to provide phenomenological/experiential account of the mental health challenges, notably, experiences of absence of empathy, social support, and depression in pregnant adolescents. In addition to understanding the experiences of these young girls, aside from their primary caregiver such as mother, we have also interviewed the social workers—both community health workers and health care workers—and maternal and child health (MCH) center staff nurses to probe their understanding of the adolescent’s distress, their engagement with adolescents, and identification of barriers. In addition, this study seeks to understand health service gaps and challenges from health providers’ perspectives (including both specialist and lay health workers.

The paper develops four strands of evidence from qualitative interviews that address the following specific concerns.

1. To understand depressed pregnant adolescents’ experiences and needs (given the high prevalence of depression [55–80%] in this population (Osok et al. 2017, unpublished) (sample 1)
2. To understand pregnant adolescents’ needs from their caregivers’ perspective (sample 2)
3. To understand pregnant adolescents’ needs overtime (whether the challenges and needs change after giving birth) (sample 3)
4. To understand pregnant adolescents’ needs from health centers’ staff perspective (including health professionals and community health workers (CHWs)) (sample 4)

Our paper contributes to perinatal health of adolescents in Kenya in the following ways: highlighting the mental health-, family support-, and health services-related challenges encountered during the pregnancy and after delivery of the baby. In our interviews with a few caregivers, we demonstrate the complexity of competing needs and limited resources the family can lend to the adolescents. The interviews with the health care workers and health service providers highlight the knowledge and resource gaps at the facility level in dealing with the pregnant adolescents and the challenge in prioritizing their maternity and mental health needs.

Despite mounting evidence, mapping the public health burden of pregnancy in adolescence and the use of unskilled primary care taskforce to provide mental health care to young girls and women, we still have enormous barriers (knowledge, motivational, social, economic, and cultural) as well as translational challenges in providing care to adolescents in perinatal spectrum. In this paper, we adopt an exploratory systems approach to addressing the problem of adolescent pregnancy and mental health issues from multiple vantage points: unraveling the complex nature of the problem (pregnancy and depression in adolescence), complexity of the stakeholders involved (MCH nurse in charge, CHW, nurses in other programs at the well child center), and complex needs of the adolescents (health, economic, social, and family support, interpersonal, nutritional, etc.). Our goal was to map multiple stakeholders’ views on complex problems, awareness, and engagement of the key stakeholders in understanding adolescent pregnancy-related mental health distress.

## Methods

### Design, Setting, and Participants

This is a grounded theory utilizing multi-stakeholder perspectives to understand challenges and barriers experienced by pregnant adolescents. We used grounded theory approach as it does not presume existence of a theory from the outset instead uses the participants’ data to create meaningful categories and theoretical thrust (Strauss and Corbin 1990). This study was carried out in the field setting of two Nairobi city council maternal and child health centers located in Kariobangi and Kangemi neighborhoods. We divided the samples into four to highlight the different stakeholders we engaged with (also described above). Our *first sample* was of pregnant adolescents who were screened for depression using the Edinburgh Postnatal Depression Scale (cutoff of 13 or more being the criterion for being depressed). Using this cutoff, we found that eight out of a sample of ten were found to be depressed. These adolescents were from 15 to 18 years of age, and four currently lived with their partners. Our first sample had participants who interviewed at the Kariobangi health center. The *second sample* was of caregivers including partners of the pregnant adolescents who were probed about their everyday living, housing, food security, and interpersonal challenges. We interviewed two caregivers from Kariobangi and four from Kangemi health center, and these were semi-structured key informant interviews (KII). Three of the interviews were with mothers who accompanied the adolescents while one was with a male partner. Our *sample three* had two groups of adolescent mothers: we had five new adolescent mothers followed up from sample 1 (Kariobangi site). We interviewed four new mothers from Kangemi MCH site for key informant interviews, and in sample three, we included a focused group discussion (FGD) with 13 new adolescent mothers at Kariobangi. The purposes of FGDs were to identify common themes and to identify cross-cutting issues that needed further probing from the participants. *Sample four* comprised of health service providers and community health workers, making it a total of 20 (13 from Kariobangi and 7 from Kangemi health center). We carried out one FGD with CHW and another FGD with health care providers.

The study received ethical approval from the KNH/University of Nairobi IRB (no. KNH-ERC/A/411 (P520/08/2015) and was given authorization to proceed by the county health directorate and by the Director of Health Services. The nurses in charge of both facilities were contacted and sought permission from to conduct the interviews. We ensured that we did not disrupt the flow of the clinic in seeking adolescent or their caregivers or the MCH personnel’s time. Caregivers gave consent for their adolescent daughters/partners to participate, and we sought assent from the adolescents themselves before the interviews or data collection began. We sought consent from the adolescent in instances when she was living by herself or was given a status as an “emancipated minor” (MoH, Kenya 2015). None of the participants we approached refused participation. We offered adolescents’ milk and bread and paid for their transport (200 Kenyan shillings) as the interviews did take time away from their routine, and we also carried out screening for distress experienced by the adolescents using the EPDS and provided referrals to Kenyatta National Hospital’s Department of Mental Health and at times to the psychiatry clinic which runs at the MCH facility through the cooperation of the University of Nairobi/Kenyatta National Hospital.

### Study Variables and Interviews (Time, Type, and Duration)

We developed an interview template which acted as a guide to the work and touched on a set of cross-cutting domains, and we included few stakeholder-specific questions in our interviews. Key stakeholders were purposively selected to elicit first-hand information about challenges experienced by the adolescents. The interviews were conducted from November 2015 to January 2016 at the health care facilities. The interviews were audio-recorded and typically lasted 20–40 minutes. The average duration of the interviews was 22 minutes. These were subsequently translated and transcribed into English. Table 1 provides thematic domains of the interviews including the number of participants interviewed. In addition to the first author, two research assistants who were fluent in Kiswahili and the local Kenyan dialects were part of the interview. A small cubicle was provided by the health facility to conduct these interviews. We interviewed the adolescents separately. Sample 1 and 2 interviews were key informant interviews and therefore done on one to one basis. In samples 3 and 4, we utilized both KII and FGD to generate individual impressions, and then, our effort was to triangulate these responses in a group context. In sample 3, we interviewed a few adolescent mothers individually who had delivered babies (whom we had met in sample 1), and then. we held FGD with new adolescent mothers whom we were meeting for the first time. The adolescents from sample 1 were mostly not available due to their varying circumstances. As this is a difficult group to locate at clinic level, we wanted to utilize the opportunity to learn more about their challenges once they delivered the babies. In sample 4, the MCH staff and health workers were interviewed individually and then invited for FGD as we found them eager to know about each other’s’ experience and wanting to exchange notes about the challenges in working with this group. We used the FDGs to clarify individual interview questions but also to explore what they knew about the adolescent mental health issues and how can they lend more support to the adolescents.

### Data Analytic Approach

The interviews were audio-taped and after, their transcriptions were read by a team of three individuals. The first author read each transcript and developed themes per interview. These interviews and the preliminary themes were then reviewed by research assistants who were involved in interviewing the participants. One of the two research assistants is a clinical psychologist and the other is a public health researcher both trained in qualitative research methods. Both research assistants divided interviews between them and reviewed initial coding and provided further inputs. The team then triangulated collated interview themes from the transcripts and these were then categorized into core themes enlisted in Table 2. Two sample interviews of each of the stakeholders were then read by the other members of the team and Table 2 findings are discussed at length with each author to assimilate core findings. At each step in this research, the idea was to stay as close as possible to the “real/lived experience of the participant” and to recount adolescents’ experience, support mechanisms available, and various barriers before them. These findings were deliberated with the pediatrician (DW) and psychiatrists (CO, SKN, and JN) for further refinement. Each theme generated was discussed between authors and the evidence for the sub-theme detailed and deliberated. Authors MM and KYH were consulted once themes were firmed up to write the discussion section. Each of the authors gave comments on the results and discussion—it was important to ensure that we were true to the themes expressed in our discussion. The first author who took lead on this paper ensured that the team had gone through interview transcripts to get a sense of the data before results were put out. Tables 2 and 3 are key implementation-related barriers and health services’ focused case illustrations of interviews with community health workers and the MCH staff.

## Results

The interview themes and corresponding vignettes extracted from the interviews are summarized in Tables 2 and 3. Table 2 highlights core themes that the pregnant adolescents shared along with corresponding vignettes. These themes kept repeating themselves by the time the third sample was interviewed which indicated that we had reached the point of saturation. Table 3 highlights the problematics of connecting adolescents and their families to primary care by CHWs. Table 3 presents certain service and knowledge gaps highlighted by the MCH nurses.

## Depressed Pregnant Adolescents’ Experience and Needs (During Pregnancy)

### Adolescents’ Perspective

These can be grouped into five themes: (1) social stigma, (2) lack of emotional support, (3) new life adjustment stress, (4) poor health care access especially to MCH clinic assistance, and (5) future planning. Five themes stood out clearly from the interviews with adolescent participants.

#### (1) Social stigma

The first core theme centered on the experience of pregnancy and the burgeoning negative self and social stigma it had caused to them and their families. Young girls expressed feelings of isolation, loneliness, stress, and depression as common lived experience around pregnancy. There were some girls who articulated that this meant an end to their education and the need to secure a livelihood and support their child, while others were in denial and disbelief about being pregnant and said they would put up the baby for adoption. The attitude as well as the lack of support from their male partner and/or boyfriend added more pressure and acerbated feelings of despair and sadness.

#### (2) Lack of emotional support

The second theme we identified was the lack of emotional support from the partner/boyfriend. Most pregnant/parenting adolescent girls were dealing with the problem alone and/or were abandoned by their male partner. There was a feeling of disdain and trauma at the male partner having denied the baby being theirs, and 9 out 15 adolescents said their boyfriends denied any responsibility for the state the girl was in. A few that lived with their boyfriends or with his family felt that they were better supported. However, three adolescents talked about not receiving enough money/financial support and physical and health care, and talked about domestic abuse from their partner. Social support from the primary caregivers such as mothers and grandmothers was critical to the pregnant/parenting adolescents’ well-being. The caregiver’s viewpoint and attitude played a vital role in the treatment of the adolescents. Three participants we spoke with were alone and were severely ostracized by their parents; they felt that they could manage the situation on their own through working and fending for themselves and the baby. Two of out the three girls did not have any support from their boyfriends. One of these girls mentioned that she would try to reach out to the CHWs and the health facility to get medical support and search for a job.

#### (3) Stress based on new life adjustments

The third theme focused on life stressors and pregnant/parenting adolescent’s worries around the pregnancy and childrearing, and additional adjustments that would bring to themselves and their extended families. The primary focus of these themes was among adolescents’ in advanced stages of pregnancy where the primary concern included securing (or obtaining) material resources and preparation for the arrival and care of the baby. Most of the girls came from resource-deprived households, who had high food insecurity, including not having enough to eat them; therefore, providing for a newborn and their own needs exacerbated adolescent’s worries about self and child care. The mothers expected the pregnant/parenting adolescent to find work while she would look after the baby. As we studied our data, some of these burdens became a source of continuous and serious psychological distress leading to anxiety, depression, and other kinds of mental health problems. These life stressors have to be looked at in an intergenerational manner as the family and the pregnant adolescent are affected and caught in the rigmarole of poverty and marginalization.

#### (4) Poor health care access

The fourth theme focused on the health services gap that exists in Kenya and LMIC around child/maternal care and resources, as well as the attitudes and beliefs of MCH service providers. The adolescents felt neither understood nor well received in the facilities. Most of the girls needed this state-sponsored free service but put up with a lot of derision and shame. While we did not examine the negative reaction to the health facility in details, it could be that social isolation and stigma associated with early pregnancy adds to the doubt about being well received in the health care. The adolescents also mentioned that service users like themselves felt that they were mostly berated and scolded by the nursing and health facility staff. In our reading of the interviews, attitudes, beliefs, and practices of service providers contributed to the distress and marginalization experienced by the adolescents. Poor health care access was not only about limited adolescent and girl child-friendly services but also about a certain dismissive and punitive attitude of the providers. Some of these adolescents were being cast out of the health care cascade by their negative and unhelpful experiences by the health service providers.

#### (5) Planning for the future

The last dominant theme was about finding oneself without any support which necessitated planning the immediate future and post-pregnancy life, again by oneself. The pregnancy for most pregnant/parenting adolescents meant that they would have to plan for the future alone almost as a single parent. Most of our participants said that it was the end of their education and they would need to find employment in order to look after themselves and the baby in the long run. Only one adolescent who had good parental support mentioned that she would return back to school and carry on with her studies as long as she can. However, for the rest, educational opportunities had prematurely ended with the pregnancy, as the same resources would be diverted to the birth and care of the baby. Some of the adolescents discussed coping strategies and efforts to deal with their distress by engaging with their mothers to look after their baby. Some of them felt very committed to returning back to school and believed that with their families support, they could actually do both: going back to school and bringing up the baby with the mother’s assistance. However, we had a number of mothers whose own growing families (with the adolescent’s father or a new partner) were clashing with the adolescent’s pregnancy. They felt that it was going to be very challenging addressing food and other material provisioning for everyone in the family. There was also a feeling of emotional and physical fatigue among the mothers who recounted that they cannot take up the dual role of a caregiver and breadwinner for the family. Two adolescents who had estranged ties with their families and were sex workers talked about reaching out to the community health workers and social workers in trying to solicit support both for the baby and other provisions for themselves. Most of these girls appeared distressed, and two young girls (around 16 years) appeared physically unwell and extremely malnourished with no money for transport back home from the health facility. Almost all participants used the words “depressed” [Kiswahili idiom] and “stress” [Kiswahili idiom] and exhibited feelings of isolation since the news of their pregnancy. Two participants had not shared their pregnancy with their immediate family members, with the exception of their mother.

### Caregiver Experiences

Most of the caregivers accompanying the adolescent were mothers and were interviewed along with the pregnant adolescent. Even though feelings of anger and sadness brimmed up, the mothers were sympathetic and reasonably caring towards the girl. In five cases, the adolescent’s mother recounted during the interview about similar situations that the mother herself had an early pregnancy and felt “chastised” by the daughter’s early pregnancy too. In all of these cases, the young men had disengaged and refused any responsibility in the caregiving process, which made the mothers very upset. Almost all caregivers we spoke to talked about additional burden of food insecurity and greater marginalization within family due to the adolescent’s pregnancy. Two of these mothers considered putting the babies up for adoption. Three mothers alluded to plans of sending the daughter away to her grandmother’s rural home away from Nairobi after the baby arrives. Two mentioned that they plan to send the adolescent away to other older relatives after they finished their exams so that the girl could deliver and stay there. Three mothers mentioned that they would persuade the boy’s family to “own up “ to the baby and look after the child because it belongs to them as well.

## Parenting Adolescents’ Experience and Needs (After Giving Birth)

### Adolescents’ Perspective

Nine of the adolescents who were followed up by from sample 1 shared that their life stressors persisted. The main issue revolved around the psychological burden of being a single caregiver and the absence of a partner’s support. Pregnant/parenting adolescents felt that they were unprepared for the challenges of childrearing and parenthood. Stressful life adjustments such as rapid body changes associated with maternity, absence of nourishing food, lack of other supplies such as nursing and sanitary pads, and other provisions for the baby were their main worries. The access to the health care facility was another challenge given that there was very little orientation given to infant caregiving, feeding practices, and prevention of illnesses. The absence of MCH outreach services in the community with easy access, as well as organizing transport to and from the health facility, was a big concern. When medications were prescribed that were expensive or not freely available, it contributed additional stress to the adolescents. In the FGDs with 13 adolescent mothers, they stated that some of the new parenting challenges included the absence of infant feeding and caregiving information. These mothers were also very vociferous about feeling the need to be economically independent and provide the best possible care for their babies. This included negotiating with their own partners, parents, or relatives for additional resources or money, which was a difficult process, given the already limited resources of the households.

Another dominant theme was their own unmet needs whether it was around food, health care, or material provision post-arrival of the baby. Their own nutrition or care became absolutely secondary. The participants talked about not finding solutions to their psychological stress or sometimes low mood conditions, and in this regard, gaining some support would significantly improve their quality of life and functioning.

### Caregivers’ Perspective

We had limited access to the caregivers after the baby was delivered, as most of the adolescent’s mothers came on their own after the baby was born. We noticed that, mainly, mothers visited the facility; perhaps, this was to minimize the time spent away from other caregivers who were responsible for work, and queuing up in the facility could take almost half of the day. Data collected from the FDGs revealed that finances were limited among the families that sought resources from the center, and by utilizing the center was one way to get additional resources for themselves and their baby.

## Needs and Challenges in Providing Service in Maternal and Child Health Services Context

### MCH Service Provider’s Reactions and Thoughts About Pregnant Adolescents

The nurses in charge we spoke with were aware of the fact that standard treatment offered at the MCH facility was not adequate to engage with pregnant/parenting adolescents. They were also aware of the stresses the adolescents went through and shared poignant stories about the disengagement of Kenyan men especially young boys from pregnant adolescent’s situation. One interesting viewpoint that emerged from these interviews was awareness or knowledge of sexual health, such as protection during sex, human development and general health of their bodies, and the social stigma and implications of early pregnancy. At both health facilities, the nurses affirmed that often, these girls are unaware and distressed about bodily changes during pregnancy and most are poorly nourished. Their role included general health and psychoeducation around pregnancy on how one should prepare for it. The nurses alluded to the lack of social support from their immediate and extended families, especially for those pregnant/parenting adolescents residing in rural areas, who face additional challenges with access to services and lack of schools or schools that were not receptive to early pregnancies, therefore becoming a site for further discrimination and alienation for the youth. The nurses also pointed to the “naïveté” of the adolescent in understanding the broader implications of having a baby and felt that the support they offered was limited and that communities need to provide additional support.

The issue of negative peer pressure and negative familial role models within a community that fosters unions between girls from poor households who get married to older men and sire children early to secure a comfortable life has become a cultural norm and an aspiration for several girls from resource-limited families and communities. These pregnant/parenting adolescents evocate this as a success story to be emulated; however, many are unable to achieve this and most suffer with the realities of caregiving in resource-poor settings. During the interviews, the MCH staff raised the issue of parenting and the absence of mental health programs (both treatment and preventative) in addressing issues that arise with early pregnancies. They also highlight that there has never been any programmatic focus on parenting or mental health in their training programs. While they felt they knew very little about mental health, most understood the family’s plight and the distress the adolescent was in, and could potentially intervene if there was training on identifying and intervening in high-risk cases.

### Community Health Workers and Their Engagement with the Pregnant Adolescent and the MCH Staff

The CHWs in both facilities included at least one male, and each health worker reiterated that the issue of adolescent pregnancy was a public health problem. CHWs in both facilities reiterated that adolescent pregnancies and its individual and societal consequences was a public health concern in Kenya. They further emphasized the challenges in engaging male members of these communities and stressed that the lack of education and the resources to provide basic education around this topic was limited, which contributed to some of the inequities and its after effects around issues with early and adolescent pregnancy and caregiving. CHWs were aware of the difficulties that pregnant adolescent encountered at all levels, and they served as a vital link between the adolescent and the health facility, in obtaining information and access to care. Often times, they would reach out to families where an adolescent was pregnant or if a family requested their support in assisting and providing linkages to the facility and services. The CHWs spoke about the health providers not giving enough attention to very needy girls and sticking to their numbers without concern for the well-being and circumstances surrounding the pregnant/parenting adolescent. However, most of the health workers agreed that the facility had become more porous and open to providing services to this population. They noted that more was needed in terms of readiness to embrace a humanistic and professional approach. One issue that repeatedly appeared during the interviews with the CHWs was the absence of any formal acknowledgement, recruitment, or continuous professional training from the Ministry of Health. In the context of adolescent and early pregnancies, the burden of domestic violence, HIV, and poverty in the communities, the CHWs can be front-line providers of preventive and intervention services; their role currently bridges gaps between the school, families, and health facilities. Two male CHWs, we spoke to, talked about engaging with boys and understanding their fears when a girl becomes pregnant, and suggested the need for better sex education and greater involvement of men in MCH programs. The CHWs were also concerned about the increase in systemic barriers as a result of the MCH worker’s distance and undermining their importance as new fathers. The absence of social workers who are paid professionals also widens the implementation gap.

## Discussion

From a system science framework, our exploratory study tries to offer some insights into a qualitative agent-based modeling approach (Brownson et al. 2014). We have offered “bottoms up” views from these multiple stakeholders in understanding depression, mental health, and social services for pregnant adolescents. The various concerns of key stakeholders intersect in the area of access to health care, material, and social supports, which are often lacking or inadequate to meet the needs of pregnant and parenting adolescents. Through examining their differential concerns and positioning the discourse around mental health of the pregnant adolescents, we highlight existing gaps within the social as well as the service share. Our paper highlights multilayered concerns of an adolescent who inadvertently becomes pregnant and is surrounded by structures that in principle do not provide the necessary supports and remain moribund or hinder access to appropriate resources. The inner-system challenges such as lack of communication; cultural, systemic, financial, and policy-related constraints between stakeholders; the complexity of pregnant adolescents; and their dependence on various institutions like family, school, health, and community resources make the problem multidimensional. Additionally, our data espouses five core themes that are intrinsic to the health and well-being of pregnant adolescents. The themes that emerge include (1) social stigma, (2) lack of emotional support, (3) new life adjustment stress, (4) poor health care access, and (5) future planning. It is important to note is that in the interviews with stakeholders such as caregivers, especially the mothers of adolescents, MCH workers, and CHWs, the same themes emerged pointing to an awareness of these challenges. The main difference in the ways of stakeholder’s perceptions of pregnant adolescents and the associated psychological burden that it causes was that they all talked about the absence of support and institutional investment in caring for the adolescent and their baby. Perspectives from the health care workers at the facility and community health workers focused on the lack of training and enhancing training around maternal/child health, and adolescent development. Additionally, they talked about developing local capacity to intervene around issues related to mental health and the specific health and social needs of adolescents in the perinatal spectrum.

Currently, there is a lack of specific interventions in the region that addresses the needs of pregnant/parenting adolescents. We suggest the development and implementation of evidence-based interventions that incorporate social, behavioral, and economic components. Furthermore, we argue that interventions need to be culturally sensitive, embedded within the local system to highlight social roles and family order, leadership, and policy gaps, as well as the communication flow between different stakeholders. The existing health and social disparities of pregnant/parenting adolescents in Kenya make implementation of existing interventions challenging, noting existing risks such as exposure to violence, HIV/STIs, food insecurity, school drop-outs, poor skills enhancement, and other adversities. The context of early pregnancy adds to the existing burden of diseases and disability-adjusted living years (DALYs) (Odejimi et al. 2011).

Systemic and economic issues such as poverty serve as both an antecedent and consequence of adolescent pregnancy. Lack of food, inadequate housing, school drop-out, and compulsion to engage in income-generating activities all increase adolescent’s vulnerability to transactional sex, sexual experimentation, and early marriage, which often leads to unintended pregnancies, STI/HIV, and continued poverty (Juma et al. 2013). A significantly higher percentage of teenage mothers and their partners had lower educational achievement compared with adult mothers and their partners. Adolescent mothers were more likely to be economically disadvantaged than the adult mothers (Taffa 2003; Odejimi et al. 2011). Adolescent pregnancy abruptly limits and ends girls’ potential because they are taken out of school to be mothers. Children of mothers with little education are less likely to be educated (PEPFAR/UNAIDS 2007; UNFPA 2013).

Recent studies in global mental health suggest that task-shifting/sharing can be effectively used with lay community health workers in LMIC (Petersen et al. 2012; Chowdhary et al. 2014), and yet, the interviews expose us to absence of any empowerment or skills development in CHWs working in both facilities. In addition to this, there is also an absence of a mental health component or training in the MCH worker program. We know how mental health and behavioral aspects inform maternal health-seeking behaviors, infant feeding, and adherence to key medications, and yet, there is paucity of integrating a comprehensive care component in MCH facilities (WHO 2006, 2007, 2009). Ultimately, the effectiveness of task-shifting/sharing appears to be contingent upon lay counselors participating in programs that include guidelines on training, supervising, and sustaining mental health treatment delivery such as the apprenticeship model described by Murray et al. (2011) and Kotte et al. (2016). There are large gaps between evidence-based practices, community services/resources, and primary care services (Brownson et al. 2014; Brookman-Frazee et al. 2015; Jenkins et al. 2013). There is a need to strengthen social work practice and offer a better integration of social work services in primary care setting (Parker et al. 2014; Muga and Jenkins 2008). During the MCH facility interviews, we found that there were no social workers attached to the facility. Therefore, both the community and the health care staff relied on poorly trained community health care volunteers who were well intentioned and accepted by the community though their training was highly limited.

### An Underserved Population: Adolescent Girls in Kenya

The most striking finding of our qualitative inquiry is that the MCH treatment as usual model cannot be used to offer treatment to adolescents who often are suffering with depression and have other health concerns. The facilities would need to be differentiated and provide services to meet the specific needs of adolescents. The other issue which we did not assess but needs further analysis is the assumption by stakeholders of the “naiveté” of the adolescent in becoming pregnant. The interviews suggest that the girls entrusted these “boyfriends/men” (most took the relationship seriously) as a partner and a future breadwinner and caregiver, either as a way of becoming independent/individuated or as an escape from abject poverty of their parental homes. The young girls we met used descriptive words to convey their emotional states in a more articulate way than the nurses in the health facility did. It appeared from the adolescent interviews that they knew precisely what they were doing, but did not know how or whom to solicit support from (both concrete and emotional).

Caregivers were mainly mothers, and if this was a younger mother who also started off the reproductive journey as an adolescent, through these interviews, we learnt how much families were negatively impacted as a result of this development. The pregnant adolescent became a constant reminder to her mother of her own failure. If this mother had remarried, both the adolescent and her mother would have to negotiate responsibilities and involvement in the care of the baby. There were instances when the mother had a toddler herself and struggled with how she would look after the adolescent’s baby and manage her other work and family commitments. Despite adolescents’ mother being the most dependable and supportive of all caregivers around her, she herself was often overburdened and stigmatized by the extended family. The pregnant adolescents were aware of how disappointed and angry their mothers felt but knew that they would try to help their daughters. Some girls mentioned that the mother would try to garner support from the father, and even if the relatives would be unsupportive, the pregnant/parenting adolescent would be protected by her parents.

### Potential Barriers to Providing Care: Some Caveats

The absence of any mental health training, educational elements around parenting, working with adolescents, or delaying early pregnancy in both MCH staff and the CHWs presents us with great challenges in the Kenyan context. The MCH staff wanted to offer support group although they had no mandate or even resources to make it happen. During the interviews, we probed if the ongoing support group for the HIV program could be used as a template to engage with the adolescents, and the nurses did think that could be possible. However, when one of the researchers probed the relevant nurse in charge about what they did in this HIV program called “mentor mother,” she replied that they provided psychosocial support. Further probing what psychosocial support meant, the nurse repeated the same that it was “psychosocial support” and this banter carried on until nothing new came out. We also got a sense that the awareness of mental health problems was particularly poor among the MCH staff even though the personnel go through nursing training. The phrase support group is equal to psychosocial support, to psychosocial support, to nothing. Psychosocial support becomes a frozen metaphor or a buzzword, not a meaningful or cogent concept to the MCH staff. This situation reflects an absence of training, guidance, or apprenticeship in order to enhance basic health and mental health skills, as well as an enhancement of translational skills where MCH staff and community health workers can both be trained in addressing mental health or psychosocial needs of vulnerable populations such as pregnant/parenting adolescents.

The power inequity between the MCH staff and the CHWs and the absence of a buffer group called social workers who are in the formal system meant to bridge the gap is palpable. There is also a need to include counselors whether lay or trained (even student volunteers), clinical psychologists, and nutritionists into the system in order to ensure that some of the task-shifted elements can be implemented accordingly and function as intended. The dismissiveness of MCH staff towards patients once their daily quota is completed or towards CHWs requires focused and systematic redress. One way to address some of these issues is to have a follow-up on pregnant adolescents who visit the MCH facility by a nurse in charge and provide support to CHWs, which is vitally important. Carrying out a risk assessment of the adolescent’s family condition and her support mechanisms is something that can be done at the community levels; this can address the health and medical needs, as well as bolster social connections. Furthermore, child welfare services’ involvement in this process is important.

## Figures and Tables

**Table 1:** Participants, their numbers and location, and core areas explored in the interviews

Participants, *N* and location	Interview type	Core areas explored
Sample 1. Depressed pregnant adolescents, (*N* = 8 from Kariobangi site)	Key informant interviews	Their personal/emotional experiences, relationships, and circumstances of pregnancy, family and social support available, interface with health facility, plans for future
Sample 2. Caregivers of pregnant adolescents (*N* = 6 from both sites)	Key informant interviews	Their reaction to the pregnancy, resources available to support the adolescent, plans for future for adolescent and the baby, support from community and CHWs
Sample 3. (i) Follow-up from sample 1: new adolescent mothers who gave birth, (*N* = 9 from both sites); (ii) new adolescent mothers: (*N* = 13 from Kariobangi site)	(i) Key informant interviews (ii) One focused group discussion	Reflecting on their experience of pregnancy, evaluation of support available from family, partner, health facility, and CHWs. Knowledge about adolescent pregnancy, their mental health problems, referrals and support available to them and their caregivers, challenges in working with this group
Sample 4. HW, CHWs, MCH staff/medical staff, (*N* = 20 from both sites)	Two focused group discussions	Experience and knowledge about adolescent pregnancy and their mental health problems, referrals and health facility support and engagement, challenges in being a bridge between families and the health care system

**Table 2:** Multi-stakeholder views on adolescent pregnancy and depression

Sample 1: *Interviews with 8 depressed mothers and 2 non-depressed adolescents.*			
*Given high prevalence of depression among pregnant adolescents (80%), our 1st goal was to understand these depressed pregnant adolescents’ experience and needs*			
Study samples and key interview objectives	Core themes		Corresponding vignettes from interviews
*1. Adolescents’ experience with pregnancy* Personal experiences (positive or negative) Challenges (economic, social, medical) Psychological well-being (experience with depression particularly) Coping mechanisms *Coping mechanisms and positive thinking demonstrated* *2. Reactions from boyfriend and other related members (friend, relative, neighbors)*	*Overall, none of the adolescents felt it was a positive experience.* It was overwhelmingly difficult experience only the number of adversities varied from person to person. Negative experiences were as follows: (a) *Lack of emotional/relationship support*: Majority of depressed pregnant adolescents did not receive needed support from boyfriend or family members. The most painful thing was that the boyfriend/partner vanished from the sight once a girl was pregnant. Two girls were working as sex workers and in a disconnected and distant voice mentioned that this was the fate of their lot. (b) *Social stigma* The main challenge was stigma, unacceptability by the family starting from the male figurehead. For most girls. it was a negative experience filled with shame. For two adolescents, the partners were neighbors who rejected them when they learnt about the pregnancy. Even for the individual with family support (*n* = 1; caregivers help bring up the baby and send her to school), the pregnant adolescent still felt ashamed and demotivated. (c) *Life adjustment stress* Food and financial insecurity, and end of education and a struggle to deal with pregnancy and baby while thinking of finding a job. The immediate implication is that the girl has not been focusing on her studies/work but the pregnancy is a punishment for her transgressions. (d) *Health care access* Apathy but it cannot be avoided as the girls or their families cannot afford healthcare. At times. CHWs help map the communication gap between the nurses and girls/patients *Adolescents also displayed positive thinking and useful coping mechanisms and strategies aiming to reduce their stress* (e) *Useful coping strategies* Soliciting maternal support so that the baby can be looked after and the adolescent can find work. Problem solving and staying positive by focusing on the baby was a potential support and a loving being (in Kenyan cultural imagination the baby is highly valued). (a) *Partner/boyfriends reactions*: this is mostly negative. However, we had one partner who was very supportive and aware of his role (b) *Support is often mainly from the mother* Among 8 interviewed depressed adolescents, 3 were accompanied by their mothers during prenatal visit, and 1 was accompanied by partner. Most girls said that the boyfriends denied that the baby was theirs even when the adolescent’s parents pursued the boy in owning up responsibility. The mother/other maternal figures are a little tolerant but the father and his family use this opportunity to caste a dark shadow on the irresponsibility of the adolescent’s mother in parenting her. (c) *Occasional family rallying support to look after the baby* Among those with family support, one mentioned that her parents had accepted to help, bring up the baby and send her to school; Two were living with the grandparents in the rural homes where they got pregnant and were relocated to Nairobi to be looked after by their mothers		“I feel embarrassed all the time and also feel that I have let them (family) down.”-15 years old “Thoughts and anxieties are many and I am not as happy at home but I am fine at work.”-18 years old, married, domestic violence and lack of spousal support) “I don’t know whether I am depressed the way you describe it but I regret a lot (crying again). I went to a bridge at home and wanted to throw myself because my grandfather is very harsh and an aunt told me it is a sin.”-15 years old—full term—almost 9 months pregnant “We used to get along as neighbors. One day I went to visit him, he convinced me to do that thing and I agreed and see the consequences and now I am stigmatized for life.”-15 years old “I do not have hope. I have many dark thoughts because we do not have money and I do not like insults and noise from his (partners’) mother. I do not sleep well, eating is tough sometimes but he is trying and I am trying.”-17 years old, married “I am the only one who reached class 8, the rest of us have not been to school. He (the father) had promised to take me to school. Earlier on, I was staying with my sister but food was always a problem for her and her children and me were struggling. She advised me to remove the pregnancy that’s why I decided to go to back to my home. I have been chewing miraa (khat) that eases the pain.”-16-year-old pregnant adolescent’ high scores on EPDS for depression) “I have no one to look after me and girls like me have to find their own way. I will talk to the social worker and find some help to start a business or some food support from them. I can’t do the same thing now (sex work) and if I can’t manage I will give up the baby to an adoption home.”-17-year-old pregnant adolescent “Once you make a girl pregnant, you have to support her. Both of us are responsible for this. I know men leave girls/women alone. I am there to support my partner”-boyfriend of an 18-year-old pregnant adolescent “I have had a very hard time since I was in class 8 preparing for my exams. I hid it for 5 months but it started to show. The boy has denied the child.”-15 years old “We continued as friends until I missed my periods for 6 months, I knew I was pregnant. I told him and he said I have been with other boys in our school.”-15 years old. “I am thinking that when she gives birth, I will take the baby to the father, leave the baby on their doorstep. I do not have a job. I am dependent on my husband. We have 3 children; the last 2 were twins 10 months old. It’s been hard living with her and this pregnancy. I cannot take care of another child.”-15-year-old mother
Sample 2: *The 2nd goal was to understand pregnant adolescents’ needs from their caregivers’ perspective. Interview data for this aim was collected from a few caregivers who accompanied adolescents during their prenatal visits.*			
*1. Caregivers’ experience/view on their girls pregnancy* Personal experiences (positive or negative reaction) Views on challenges posed by their daughter’s pregnancy (economic, social, medical)		Following themes emerged from adolescents’ mothers who accompany child during health visit were (a) their *empathetic reactions for their daughters’ pregnancy*; even as they cope with disappointment and sadness The most poignant descriptions by the mothers were that they had babies when they were adolescents themselves and tried all their best for these circumstances to not repeat in their children’s lives. Mothers did think of this to be acts of transgressions but were more tolerant. They also lamented the choice of boys such that the same abandonment should repeat itself. The caregivers refer to their own experience of feeling let down by the adolescent, own memories of early pregnancy and feeling very let down by the family, the adolescent as well as the boyfriend. They also remember they had no friend accompanied and neither did any neighbor (b) *Concerns for new life/adjustment and life stressors for their adolescent daughters* Their main concern was for the daughter’s disconnect from education, employment or a decent future. Alluding to the price girls have to pay for something that involves men and women together The caregivers are less explicit or interested in addressing depression but more worried about provisioning for the growing needs of the adolescent, her baby. Acceptance that the situation is depressing and challenging for the young person (c) *Need for exploring solutions and preparation during current state especially those who were married and lived in parents-in-law.* Some talk about discussing psychosocial issues with CHWs to find solutions, i.e., finding extra work, medical assistance, food banks. (d) *Adolescents developing child care clashes with her own mother’s expanding family* If the adolescents mother is relatively younger and remarried her say in the family decisions is limited. One or two mothers mentioned that they had families with young kids and the adolescent daughter’s baby needs would clash/competing with her new family. No friend accompanied and neither did any neighbor Grandmothers came and once a maternal aunt came— same concerns but also worries about their own burden increasing	“I wrote a letter and took to their home, his mother was also shocked. In the letter I told them as the mother, I will inform you of her progress, but I would also like you to talk to your son so we all see the way forward. I wrote the letter because I didn’t want to talk too much out of anger. So far I have not got any positive feedback. My husband does not want me to call them.”-15-year-old mother “My daughter is my only girl, I have brought her up with the Christian value system, I just do not know what happened.”-17-year-old mother “I have realized that I am her only friend and I do not want her to be alone.”-17-year-old mother “It is so hard to get money or to meet my basic needs, I am married but we both do not work, we scrape from here and there doing odd jobs.”-17-year-old married. “We are all okay with this as parents and my mother-in-law supports me. The mistake has already happened so we are going to take care of the child and she goes back to school. I am a counselor and do this all the time for other parents and why not for my only child?”-15-year-old mother “The biggest stress is lack of money. His mother has to provide everything and these days I see it causing a lot of noise.”-17-years-old adolescent “I do not have a job. I am dependent on my husband. We have 3 children; the last 2 were twins 10 months old. It’s been hard living with her and this pregnancy. I cannot take care of another child.”-15-year-old mother “In case the doctor says the child is healthy, we would like to give him/her to a children’s home or some-body who needs a child so my daughter can go back to school because I cannot raise this one.”-16--year-old mother
Sample 3: *The 3rd aim was to understand adolescents’ experience change overtime (before and after gave birth). Qualitative data were collected from adolescent mothers (who may or may not have prenatal depression). Different from aims 1 and 2, which used individual interview format, data collection for this aim was based on first focused group and a few new mothers not interviewed earlier*.			
*Experience change overtime (before to after gave birth)* Feeling change *overtime* (when discovering pregnant and over the course pregnancy) Feeling *towards baby* (feeling change over the course of pregnancy and after gave birth) *Experience with medical care* (behaviors/attitudes towards medical staff, and experience changes overtime)		Focused group results indicated *challenges described during prenatal period maintain after giving birth* (as results described in aims 1 and 2). *In addition, new parenting challenges arise after giving birth.* *Psychological burden*—Feeling very burdened and demoralized from the relationship side *Life adjustment stress*—Coming to terms with the reality of the baby and the need to refocus on life’s priorities based on the arrival of the baby *Health service access challenges*—Overall negative to neutral experience at the health facility but one that cannot be avoided. *New parenting challenges* Worries around breastfeeding and mother’s own nutritional care. Not receiving enough money or material support from parents/partner. Feeling very low, drained out, and demotivated due to high pressure, lack of support, and material provision to care for the baby No parenting program or information readily available in the community or health center *Experience as a parent* Roles: Balance parenting with other responsibilities) Sources of support (for parenting related questions, for psychological support (for stress, sadness, depression), type of services needed) Challenges (general challenges, sources of stress, food security, relationship with partners, change roles as a parent) Parenting program experience The focused group also generated a few *suggested strategies that may help reduce adolescent mothers’ burden and stress* Finding means of livelihood to sustain this unit (sense of being a dyad) The FGDs focused on the need for the MCH staff to be more empathic and helpful. Particularly providing guidance on the kinds of changes taking place in the body, nutritional support and emotional support, and parenting support	“Problems with feeding our own selves as the food is limited in the house, some days we have to go hungry and we are told by the nurses to still keep feeding the baby as the body can still make milk.” (three 16-, 17-, and 15-year-old adolescent girls share same experience during the FGD) “We feel that the nurses/clinic look down upon us. with no food to eat, pressure to work and this new baby – no sleep, sickness of the baby and a husband/partner not supporting us….life is hard.” (two 16-year-old adolescents during FGD) “Some days I just sit and cry, I ask my mother for money to go to see a doctor she doesn’t have, I asked my partner and he chases me away. It was hard to come today to the health centre. I had to walk a lot.”-16-year-old adolescent mother. “I would wish that there should be maternity near here instead of going far …. we have to go to Pumwani or sometimes in private and I wish follow ups with the baby could be done at my convenience at my home or near to me.”-20-year-old new mother of three “Over partner coerces us for sex, my body is still sore, I don’t have the urge to be sexually active but he says I am being disrespectful.”-18-year-old new mother) “Alcoholism is my biggest pain … my partner come home he sleeps, he doesn’t get concerned about the child …. he doesn’t get concerned about me; he doesn’t to know that you’re supposed to eat such like things, yah ….”-19-year-old new mother of two “To understand how to care … care … care for baby I went at Child Fund, this time we learnt how to … how to take care of children, we were taught how to take care of those children by card, you show what is this to two months old or year old child, or below 4 years, we should know how to take care of him/her. Another thing I noticed was that the moment when the mother separates from the father, children get problems.”-18-year-old adolescent “Getting money also becomes a problem, food in the house …. sometimes like me I stay there the whole day and get may be only supper …. so if it’s the weekend they stay at home you see they have to skip lunch …. so you get stressed because the children are used to eating in school so one gets stressed.”-adolescent mother says about her family and her younger sibling, 17 years old ‘You see if a service emerges where mothers meet and then they …. a person is brought to talk to them about bringing up the baby, teaches them business, teaches them how to get money, at least you will get mothers without much stress, don’t you think that would be so much better.’-new mother, 18 years old
Sample 4: *Interviews with HW, CHWs, and MCH center nurses and program officers*			
*Health needs of parents and children. These interviews were FDGs and KII and aimed to identify the needed service and gaps (have enough resources to provide the needed service)*			
*Parental service provided in community health centers* (psychological and health services provide for parents and quality and challenges) *Child services provided in community health centers* (physical and mental health services provided for child and quality and challenges)		One key theme emerged was *adolescent mothers have multiple needs, but there is a lack of holistic service approach* *Adolescent mothers’ needs* include social support from partners (who tend to absent from the process); care for mother’s mental health; resources to obtain food for their child; parenting/child related services These girls know nothing about caregiving and are in such a difficult role as mothers Physically weak and emotionally disturbed girls and how would they attend to the needs of a new born The domestic environment generates more shame and stigma; there is also role conflict between adolescents parents about their girls’ plight. Gaps There are big gaps in thinking holistically about health needs of young parents and their children There is another gap in engaging with male partners who seem absent from the system No programmatic focus on mental health or training of CHWs on mental health, focus on adolescents Absence of networking with iNGOs to provide real resources to women and children such as food, supplements, and information. In relation to quality of maternal and child service, several gaps were mentioned This (maternal depression) is the most needed service but there’s *no expertise or even training program* available Both health professionals and CHWs agree that no services directed towards psychosocial support of parents or parenting challenges, or towards new/adolescent parents are in place. The adolescent friendly services are not there at all. The infant and child care services are minimal and do not provide relief for everyday stressful events, there are not resources or support networks available such as a breastfeeding support group Health workers say no child MH or parenting training has ever been provided to them Resource and cost: Sometimes the health facility is so pressed with time and resources that not all patients are attended to. for patients the transport costs matters so if not seen then unlikely to return again and again	“We are approachable and can link them back to the health facility.”-male CHW in Kariobangi “We offer a shoulder to cry and link these girls and women to the right places.”-two female CHWs in Kariobangi and Kangemi “What do they know about their own body or caring for a baby? They need support!”-MCH nurse in charge, Kariobangi and Kangemi “Peer pressure also determines these early pregnancies.”-CHW, Kariobangi “I would like to be able to start a support group for these mothers. I would like to find a supporter (funder) who could provide resources for these girls to meet, discuss what they need for their future, their babies and for themselves. When women got money for transport or a plate of food on their returning appointment, relaxing and talking to one another became easy ….”-nurse in charge, Kariobangi health center “Let’s take the case of HIV for example, may be the young lady/adolescent has come alone in the antenatal clinic tested and found positive …. so to take that message home it is a problem. Once the partner hears or understand or gets to know that the lady is reactive or positive; that is the end of that marriage ‘hiyo ni yako’ (that is yours) ‘you know where you got it from,’ and the man disappears. So especially in the issue of HIV, it is a problem … it is a problem. The others are financial problems, the domestic task; but in the issue of HIV it is a problem, most of the ladies are alone after realizing they are positive. Because even I talk to them when I’m immunizing their baby I am asking now you are taking treatment what about your partner, ‘he just went; he left me pregnant, he left me when I gave birth to the baby’ …. and it is all like that; it is a problem I have very many single adolescent mothers after the test.”-deputy nurse in charge, Kangemi health center “We are volunteers most of the time the money we get from are from participating in trainings and delivering to the community—these aren’t always ongoing—we rely on programs that people bring to the facility. Once we finish the clinic work we don’t stay here the whole day. In all these trainings we have done so far none has been on mental health or dealing with parenting. We use our own wisdom and knowledge in counselling women who come to us. sometimes we go to find other ways to get money— like driving taxi, doing casual work, running second hand clothes business.”-3 community health workers in a FGD, two women and one male CHW

**Table 3:** Core issues flagged off by the MCH personnel and community health workers on challenges before pregnant adolescents

Thematic domain, group that voiced these	Underlying dimensions with illustrations
*1. Pregnant adolescents and the key challenges* “What do they know about their own body or caring for a baby? They need support!”-MCH nurse in charge, Kariobangi and Kangemi “Peer pressure also determines these early pregnancies.”-CHW, Kariobangi	“Their minds have been dealing with chemistry, maths and friendships …. To direct Ones mind to the care of the baby and discontinuation of studies is the key challenge with these pregnant adolescents or new mothers” “Support from other women in their lives esp. siblings, mothers, is important …. social support is so poor as their native homes may be far away from Nairobi ….” “ think peer pressure contributes a lot because you find a young girl has a friend who is married with a kid and what of her. When she goes to try out she finds it hard to sustain since she was going so as to be the same as her friend but find that it’s not the same.”
*2. Issues of access and articulation of needs* “We are approachable and can link them back to the health facility.”-male CHW in Kariobangi “We offer a shoulder to cry and link these girls and women to the right places.”-two female CHWs in Kariobangi and Kangemi	“If they have missed appointments or struggling to make end meet, we can be approached and we link these girls back to the health facility…the community too entrusts us with responsibilities when there are complicated circumstances in life of a person” “The men have to be educated about their role in looking after the health of their children, at the moment they have distanced themselves from this and are mainly looking at food to put on the table” “We refer them to the chief, take them for free dress making or hair weaving classes. There’s someone we find to connect them to when need be. We are there to support them, when they shed tears and are inconsolable we offer a friendly shoulder” “We have to work with both boys and girls to educate them about their rights.”
*3. Various considerations around keeping the baby despite age* Kariobangi	“I told her that God has a reason because there are many who wish to have kids but they can’t so she should persevere, I think you saw how she was walking weakly (mhh …. All prompt) she is thinking very much.” “We told her that women in our culture have to be brave and bring up children ourselves. So investing in elders and community advisors like us would help her bring up the baby.”
*4. Need for support group* “I would like to offer them an ongoing support group.”-MCH nurse in charge, Kariobangi	“I would like to be able to start a support group for these mothers. I would like to find a supporter (funder) who could provide resources for these girls to meet, discuss what they need for their future, their babies and for themselves. When women got money for transport or a plate of food on their returning appointment, relaxing and talking to one another became easy …” “In the HIV context, the mentor mother program has been a great success.” “There was a support group in existence last year …. don’t know how and why it died .… probably the funding faded away.”
